# The role of neighborhood factors in the cumulative number of episodes of recurrent tuberculosis in Cape Town

**DOI:** 10.1016/j.jctube.2026.100591

**Published:** 2026-02-13

**Authors:** Eli Dearden, Frank van Leth, Marjan Molemans, Shumsonesa Abrahams, Natacha Berkowitz, Erika Mohr-Holland, Robin Wood, Sabine Hermans

**Affiliations:** a Amsterdam UMC location University of Amsterdam, Department of Global Health, Amsterdam Institute for Global Health and Development, Amsterdam, the Netherlands; b Copernicus Institute of Sustainable Development, Utrecht University, Utrecht, the Netherlands; c Vrije Universiteit Amsterdam, Department of Health Sciences, Amsterdam, the Netherlands; d Amsterdam Public Health Research Institute, Amsterdam, the Netherlands; e City Health Department, City of Cape Town, South Africa; f University of Cape Town, Desmond Tutu Health Foundation, Cape Town, South Africa; g University of Cape Town, Institute for Infectious Disease and Molecular Medicine, Faculty of Health Sciences, Cape Town, South Africa; h Amsterdam UMC location University of Amsterdam, Center for Tropical Medicine and Travel Medicine, Department of Infectious Diseases, Amsterdam, the Netherlands

**Keywords:** Tuberculosis, Recurrence, Reinfection, HIV, Social determinants, Neighborhood

## Abstract

Recurrent tuberculosis (TB) accounts for 30% of the annual TB burden in Cape Town. To better understand mechanisms behind recurrences, we assessed the association between neighborhood factors and the cumulative number of TB episodes per individual between 2003 and 2015. We used TB notification data, previously geocoded, and probabilistically linked with 2011 Census data at the neighborhood level. Individuals were grouped by follow-up time after their first TB episode: 5–10 years (FUT5-10) and over 10 years (FUT10+). Ordinal regressions adjusted for age and sex examined associations, with robust standard errors for neighborhood clustering. A secondary analysis from 2009 onward included HIV status, restricted to individuals with at least five years of follow-up. In the FUT10+ cohort, 9.6% had two TB episodes and 2.1% had three or more; this was 7.9% and 1.3% in FUT5-10, and 7.4% and 1.3% in the secondary analysis cohort (SAC). A higher cumulative number of episodes was associated with neighborhood household size across cohorts (FUT10+ aOR = 1.23 (95% CI 1.15–1.31), FUT5-10 aOR = 1.26 (95% CI 1.16–1.37), annual neighborhood TB incidence (FUT10+ aOR  = 1.13 (95% CI 1.06–1.20), FUT5-10 aOR = 1.11 (95% CI 1.04–1.19)), neighborhood socioeconomic index (FUT10+ aOR  = 0.98 (95% CI 0.95–1.01), FUT5-10 aOR = 0.94 (95% CI 0.91–0.97), SAC aOR = 0.93 (95% CI 0.88–0.98)) and HIV infection (SAC aOR = 1.83 (95% CI 1.59–2.10)). These findings highlight that neighborhood-level risk factors contribute to recurrence and suggest the role of reinfection in recurrent TB. Targeting neighborhoods with high TB incidence, larger households, and lower socioeconomic status may improve screening and reduce TB burden in Cape Town.

## 1 Introduction

Recurrent tuberculosis (TB), defined as TB disease among previously treated individuals, is common in high-incidence settings, such as Cape Town, South Africa, where 25–30% of new treatment episodes of TB are recurrences [1], [2], [3]. Recurrent TB has been associated with lower cure rates than initial TB and with increasing drug resistance [4]. Recurrence can be caused by relapse (reactivation of the initial infection) or reinfection with a new TB strain [5], which can only be determined by strain-typing. A systematic review of studies that performed this [6] reported 83% of recurrent TB to be relapse and 17% reinfection in low incidence settings, vs. 59% and 41% in high incidence settings. In high-incidence settings, relapse disease has been shown to occur mostly in the first year following treatment, while reinfection disease dominates thereafter [7]. Most studies are limited by a short follow-up time, a small sample size, and a lack of geographic representativeness, however. Individual risk factors for first TB episodes, such as age, HIV status, and household wealth, have been well established [2], [3], [8].

In population-level studies, it has been shown that each successfully completed treatment episode increases the risk of recurrence [9]. In an attempt to explain this increased risk and to identify potential avenues of intervention, we studied both individual risk factors and neighborhood characteristics associated with a first episode and a first recurrence [10], [11]. We identified factors that reflected a combination of both relapse (such as socioeconomic deprivation) and reinfection (such as increased neighborhood TB incidence). Building on these findings, this study aimed to investigate factors associated with the cumulative number of TB episodes over a long period of time, hypothesizing that risk factor patterns for this more extreme outcome could provide clearer insight into the underlying mechanisms and better inform potential intervention strategies. We analyzed the association between neighborhood factors and the cumulative number of episodes of TB disease per individual in Cape Town, stratified by length of follow-up time (5–10 years, or > 10 years).

## 2 Methods

### 2.1 Study setting

The Cape Town metropolitan area had an estimated annual TB notification rate of around 600/100,000 population in 2023, one of the highest in the world [12], [13], [14]. TB notification rates peaked at 850/100,000 population in 2008, after which the rates have reduced since the widespread use of antiretroviral therapy (ART) [15], [16].

During the study period, 101 primary care clinics provided free diagnosis and treatment of TB [9]. TB was diagnosed with sputum smear microscopy before 2013, and with Xpert MTB/RIF afterwards [9]. There have not been any major changes in diagnostic or treatment procedures of drug-sensitive TB since.

### 2.2 Study design and population

This cohort study used individual-level data from the Electronic TB Register (ETR) (2003–2015) [17] and neighborhood-level data from the 2011 Cape Town Census [18]. The case definition to be recorded in the ETR was clinically diagnosed or bacteriologically confirmed TB disease through Xpert or smear microscopy, and the initiation of treatment [19]. Patients with active disease who did not initiate treatment were not recorded in the ETR, nor were patients with drug-resistant TB, as they were registered in a separate database [20].

We constructed a cohort of individuals living in Cape Town who had their first notified episode of drug-sensitive TB between January 1, 2003 and December 31, 2015 (end of data availability, data were collected in a different data format thereafter). Treatment episodes were defined as TB notifications recorded in the ETR [17]. Because the ETR lacks personal identifiers, individuals were linked to treatment episodes using a previously developed probabilistic linkage algorithm, which was validated using manual review [9]. Geocoding to neighborhoods (referred to as “subplaces” in the Census [18]) was also based on this linked cohort [10]. To ensure an accurate count of TB episodes, individuals reporting one or more prior episodes not captured in the ETR were excluded. Neighborhoods lacking SEI data (i.e., those with fewer than 20 households) were also excluded (88 out of 923 neighborhoods) [18].

Individuals were grouped into 2 cohorts of maximum possible follow-up time since the end of their first treatment: between 5 and 10 years of follow-up (FUT5-10), and more than 10 years of follow-up (FUT10 + ). We excluded all individuals with a follow-up of less than 5 years, because of the limited time to develop multiple treatment episodes. Follow-up time was calculated as the number of days between end of the first treatment episode and December 31st, 2015. We performed a secondary analysis (referred to as secondary analysis cohort), including HIV status in a subset of the 5–10-year FUT cohort, which included individuals with their first episode from 2009 onwards and with a known HIV status, excluding participants without a known HIV status. Prior to 2009, HIV testing was not performed consistently, but this was nearly complete from 2009 onwards (92.6%) [15]. Calendar years (of the start of the first episode) were 2003–2005 for the FUT10 + cohort, 2005–2010 for the FUT5-10-year cohort, and 2009–2010 for the secondary analysis cohort. Follow-up was until the end of data availability (2015).

### 2.3 Theoretical framework

**Fig. 1:**
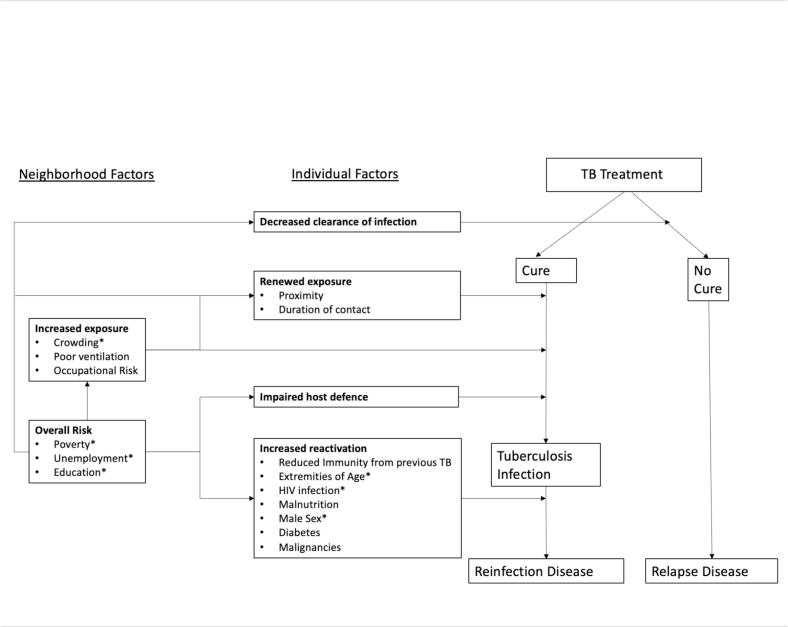
Theoretical Framework of factors associated with recurrent TB. Based on Yates et al. [8], Harling et al. [21], Lönnroth et al. [22], Bestrashniy et al. [23], Acosta et al. [24]. In the studies cited, factors such as unemployment and poverty are sometimes analyzed at the individual level; however, in our analysis, they are included as neighborhood-level variables. Abbreviations: HIV-human immunodeficiency virus, TB-tuberculosis. (*)-indicates variables available in the dataset.

Based on existing literature [8], [21], [22], [23], [24], a theoretical framework of factors associated with recurrent TB was developed to clarify hypothesized pathways of recurrence and to provide a conceptual basis for the study design and which factors to include in the multivariable analysis (Fig. 1).

The focus of this study was neighborhood factors, with inclusion of individual factors to control for individual level differences. We defined neighborhood factors as those that operate at a geographical area level, within which physical and social environmental features affect individuals’ health outcomes [25]. From the theoretical framework, variables marked with an asterisk (*) were available for this analysis and included neighborhood TB incidence, socioeconomic status, and household size. Neighborhood TB incidence and household size were hypothesized to be primarily associated with reinfection, while socioeconomic status was associated with both relapse and reinfection.

### 2.4 Measurements and variables

The outcome variable was the cumulative number of TB episodes per individual, regardless of prior treatment outcome or recurrence type. The ETR provided individual variables such as age, sex, and HIV status, while the Census provided neighborhood population size, household size, and the Socioeconomic Index (SEI) [17], [18]. Neighborhood-level variables were based on the location of an individual’s first treatment episode. Socioeconomic status was measured using the City of Cape Town’s Socio-Economic Index (SEI), derived from census data on housing, education, services, and economic indicators [26]. Due to unclear threshold justification, SEI was analyzed as a continuous variable, with higher values indicating less vulnerable neighborhoods. Mean household size, used as a proxy for overcrowding, was calculated from census categories by weighting and averaging household counts, with 10 assigned to the 10 + category [10]. The neighborhood-level TB incidence was calculated annually per 100 residents and averaged until the end of the relevant study calendar years for each cohort.

Individual-level variables included age, sex, and HIV status. HIV status was classified as positive throughout, negative throughout, or seroconverted between episodes. Age at first TB episode was used and categorized into 0–14, 15–24, 25–54, and ≥ 55 years, based on census groupings [26]. The ≥ 55 cutoff ensured adequate sample size across outcome categories.

### 2.5 Statistical analysis

We described the proportion of cumulative number of TB episodes per individual, along with baseline characteristics of each variable. Treatment outcomes by cohort and episode number were examined to assess whether the proportion of treatment default was comparable across cohorts. In addition, baseline characteristics were compared between individuals with and without known HIV status as a sensitivity analysis to evaluate potential bias due to missing HIV data.

We used a multivariable ordinal logistic regression model with robust standard errors to adjust for neighborhood clustering [27] for both analyses to evaluate the association between variables and increasing cumulative number of episodes per individual using Stata (version 16.1) [28]. The proportional odds assumption [29] was tested for each variable to be included using the Brant test [30]. As the test has been reported to be anti-conservative with large sample sizes [31], [32], an alpha level of 0.01 was used. Based on proportionality test results, we considered three models: a proportional odds model (all variables met the assumption), a partial proportional odds model (some did), and a multinomial model (none did) [33]. We did not test for confounding or effect modification for variable selection due to bias, poor interpretation, and potential negative confounding [34], [35]. All the available variables that were identified in the theoretical framework were included as *a priori* risk factors. Results for the FUT10+, FUT5-10 and secondary analysis cohorts were presented as adjusted odds ratios with 95% confidence intervals. For the secondary analysis, we compared models with and without adjustment for HIV status to evaluate whether neighborhood-level associations differed.

### 2.6 Ethics statement

The Human Research Ethics Committee at the University of Cape Town granted ethical approval (461/2015), and the Cape Town City Health Department granted permission to use the data.

## 3 Results

**Fig. 2:**
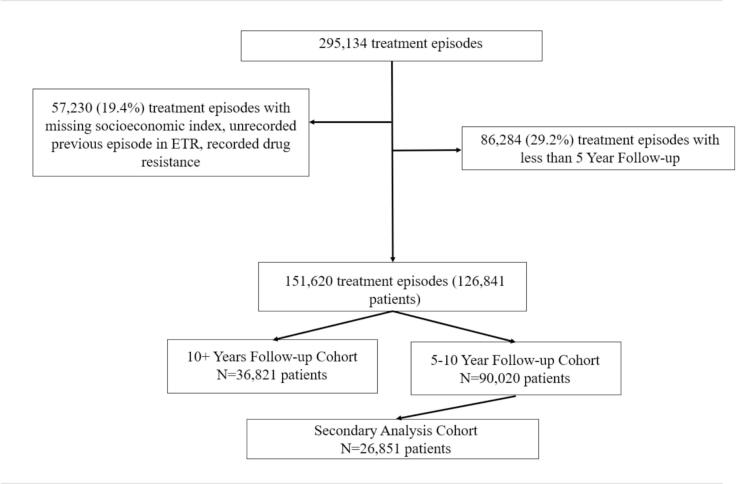
Flow Diagram for Cohort Construction. *We initially describe the total number of treatment episodes in the study period, followed by relevant exclusions. Treatment episodes were then collapsed to number of patients, such that cohort counts represent the number of unique patients. Abbreviations: ETR, Electronic Tuberculosis Register.

The construction of the cohorts based on the amount of follow-up time can be seen in Fig. 2.

### 3.1 Baseline characteristics

In Table 1, the baseline characteristics of the three cohorts are presented. Both individual level characteristics, sex and age, and neighborhood level factors, SEI, mean household size and TB incidence, were similar across cohorts. In the secondary analysis cohort, we compared the characteristics of patients with HIV status (included in the analysis) to those without (n = 1990 [6.8%], excluded from the analysis; Appendix Table 1). Almost half (44%) of those without a recorded HIV status were aged 0–14, compared to 16% among those with known HIV status.

**Table 1:** Baseline characteristics of cohorts.

	FUT10+ (n = 36,821)	FUT5-10 (n = 90,020)	Secondary Analysis Cohort (n = 26,851)
Male sex	20,175 (54.8%)	46,749 (51.9%)	13,804 (51.4%)
Female sex	16,646 (45.2%)	43,271 (48.1%)	13,047 (48.6%)
HIV status			
Remained negative	n/a	n/a	14,675 (54.7%)
Remained positive	n/a	n/a	12,088 (45.0%)
Seroconverted	n/a	n/a	88 (0.3%)
Age at first episode (Years)			
0–14	6,276 (17.0%)	15,976 (17.8%)	4,226 (15.7%)
15–24	7,720 (21.0%)	17,451 (19.4%)	5,097 (19.0%)
25–54	22,127 (60.1%)	54,889 (57.2%)	17,039 (63.5%)
≥ 55	698 (1.9%)	1,704 (6.0%)	489 (1.8%)
Annual TB incidence per 100 persons, mean (SD)	1.4 (0.83)	1.4 (0.84)	1.4 (0.84)
Mean Household size (SD) (# of individuals)	3.4 (0.7)	3.4 (0.7)	3.4 (0.7)
Mean Socioeconomic Index* with standard deviation	0.4 (0.2)	0.4 (0.2)	0.4 (0.2)

*Proportion from 0 to 1, share 1 is maximum.

Abbreviations: HIV: human immunodeficiency virus; TB: tuberculosis.

Table 2 summarizes the distribution of cumulative TB treatment episodes across cohorts. The FUT10 + cohort had the highest proportion of recurrence (defined as > 1 episode): 12.4%, followed by FUT5-10 (9.6%), and the secondary analysis cohort (9.0%). Treatment outcomes of the subsequent treatment episodes are reported in Appendix Table 2. The proportion of unsuccessful treatment outcomes increased by treatment episode, but this did not differ substantially between the cohorts. The longest median intervals between episodes were observed in the FUT10 + group, while the shortest was observed in the secondary analysis (see Appendix Table 3).

**Table 2:** Cumulative number of TB treatment episodes by Cohort.

	FUT10+ (n = 36,821)	FUT5-10 (n = 90,020)	Secondary Analysis Cohort (n = 26,851)
1 Treatment Episode	32,250 (87.6%)	81,392 (90.4%)	24,445 (91.0%)
2 Treatment Episodes	3,536 (9.6%)	7,076 (7.9%)	1,988 (7.4%)
3 Treatment Episodes	783 (2.1%)	1,196 (1.3%)	338 (1.3%)
4 Treatment Episodes	176 (0.5%)	255 (0.3%)	57 (0.2%)
5 Treatment Episodes	76 (0.2%)	101 (0.1%)	23 (0.1%)

### 3.2 Primary and secondary analysis

The regression results from the primary analysis with the FUT10+, FUT5-10, and secondary analysis cohorts can be found in Table 3. Age-category specific estimates, stratified by the cumulative number of episodes, are presented in Appendix Table 4, as they did not meet the proportional odds assumption.

**Table 3:** Adjusted Odds Ratios for an Increasing Cumulative Number of TB Episodes during follow-up.

	FUT: +10 years (n = 36,821)*	FUT: 5–10 years (n = 90,020)*	Secondary Analysis Cohort (n = 26,851)*
Variable	Odds Ratios(95% CI)	Odds Ratios(95%CI)	Odds Ratios(95% CI)
Neighborhood Variables			
Socioeconomic Index**	0.98 (0.95–1.01)	0.94 (0.91–0.97)	0.93 (0.88–0.98)
Annual TB incidence per 100 persons **	1.13 (1.06–1.20)	1.11 (1.04–1.19)	1.03 (0.92–1.15)
Mean Household size **	1.23 (1.15–1.31)	1.26 (1.16–1.37)	1.50 (1.32–1.71)
Individual Variables			
HIV Status			
Negative	n/a	n/a	Reference
Positive	n/a	n/a	1.83 (1.59–2.10)
Seroconverted	n/a	n/a	43.7 (35.1–54.3)
Sex			
Female Sex	Reference	Reference	Reference
Male sex	1.26 (1.18–1.35)	1.22 (1.16–1.28)	1.35 (1.24–1.47)

Legend: *Adjusted for Age: Age-stratified estimates are shown in Appendix Table 4.

Abbreviations: TB: Tuberculosis; HIV: Human immunodeficiency virus.

**Interpretation: For SEI, which ranges from 0 (most vulnerable) to 1 (least vulnerable), the reported odds represent the change associated with a 0.1 (or 10%) increase in SEI. This smaller increment was chosen to reflect more realistic differences between neighborhoods and to improve interpretability of the effect size. For mean household size, this represents the odds associated with a one unit increase in mean household size. For mean TB incidence, this represents the odds associated with one additional TB case per 100 population.

From Table 3, for the FUT10 + cohort, an increase of 1 per 100 population in neighborhood TB incidence was associated with 13% increased odds of a higher cumulative number of treatment episodes. For the FUT5-10 cohort, this association was slightly lower, with 11% increased odds. In terms of SEI, in the FUT5-10 cohort, a 10% increase in neighborhood SEI was associated with 6% decreased odds of having a higher cumulative number of treatment episodes. However, this association was not found in the FUT10 + cohort. In both cohorts, a one unit increase in mean neighborhood household size was associated with 20% increased odds of a higher cumulative number of treatment episodes. In both cohorts, male sex was associated with 20% increased odds of a higher cumulative number of treatment episodes compared to female sex.

In the secondary analysis, there was no association between the mean annual neighborhood TB incidence and the cumulative number of TB episodes. The lack of association with TB incidence in the secondary analysis was not due to confounding by HIV, as the association was similar in models with and without HIV status included (see Appendix Table 5). In contrast, mean neighborhood household size showed a strong association, as a one-unit increase was linked to a 50% higher odds of a higher cumulative number of TB episodes. Further, a 10% increase in neighborhood socioeconomic index was associated with a 7% reduced odds for a higher cumulative number of TB episodes. Notably, HIV was the strongest predictor of the cumulative number of TB episodes, with an almost 2-fold higher odds for HIV positive individuals compared with HIV-negative. Those who seroconverted between episodes had 44-fold higher odds (n = 88).

## 4 Discussion

We found that recurrent tuberculosis in Cape Town is strongly shaped by the neighborhoods in which individuals live, with risk emerging from a combination of community-level and individual-level factors rather than isolated determinants. At the neighborhood level, recurrent TB was more common in communities characterized by high TB incidence, larger average household size, and lower socioeconomic index (SEI).

Higher neighborhood TB incidence was associated with a greater cumulative number of episodes in the FUT10 + and FUT5–10 cohorts, suggesting that neighborhood TB incidence is a particular risk factor for reinfection; however, relapse and reinfection could not be distinguished in the absence of DNA fingerprinting [36].

Consistent with previous studies, residents of communities with lower SEI and larger households were more likely to experience multiple episodes [37], [38], and these associations persisted even after accounting for neighborhood TB incidence, pointing to other underlying drivers such as undernutrition, smoking, alcohol use, and indoor air pollution from cooking fuel [8], [22]. These findings build on prior work on single-episode TB by demonstrating how these structural conditions can sustain transmission and risk across multiple recurrences [21], [24], [37]. Importantly, the patterns varied across cohorts, with the highest proportion of recurrence seen in the FUT10 + cohort, followed by FUT5–10 and then the secondary analysis cohort. The gradient in recurrence underscores that risk accumulates over time in these settings, making early and sustained interventions critical.

At the individual level, HIV infection and male sex were both associated with a higher cumulative number of TB episodes. The strong association between HIV and recurrence is consistent with our earlier findings for a single recurrent TB episode in this cohort, as well as with numerous studies from southern Africa [3], [39], [40], [41], [42]. Although individuals who seroconverted during follow-up showed particularly high odds of recurrence, this estimate should be interpreted with caution due to the small size of this subgroup (n = 88) and warrants further investigation. Importantly, the strong association of HIV did not diminish the role of neighborhood factors, which remained independently associated with repeated TB episodes across cohorts. Even after adjustment for HIV, higher mean household size and lower socioeconomic index remained associated with a higher cumulative number of TB episodes. These results suggest an interplay whereby HIV increases susceptibility to disease, while neighborhood conditions shape the frequency and intensity of disease risk. Associations for male sex and age were consistent with those observed in the primary analysis.

Taken together, these findings shift the discussion of recurrent TB from isolated episodes to a syndemic process driven by place, poverty, and individual vulnerability. This has immediate implications for practice. Efforts to prevent recurrence cannot rely only on individual interventions; they must be complemented by approaches that target high-risk neighborhoods: those with high TB incidence, low SEI, and household crowding. The recently introduced Targeted Universal Testing for TB (TUTT) approach in South Africa involves testing high-risk individuals for tuberculosis (TB) whenever they come into contact with the health care system [43]. Target groups include people living with HIV, close contacts of TB patients, and those with a recent history of TB (<2 years). This approach could be refined by focusing its implementation in such high-risk communities [43]. In addition to being more effective, this would allow for optimal use of scarce resources and offer the greatest return on investment.

Our study has several notable strengths. It leverages population-level data with a long follow-up period, enabling the examination of recurrent TB episodes across a large, diverse urban setting. The size and scope of the dataset improve the precision and generalizability of the findings, while the linkage of multiple treatment episodes over time provides a rare opportunity to study recurrence at scale. By integrating neighborhood-level and individual-level factors, this work highlights the multilevel nature of TB risk, a perspective often missed in smaller or cross-sectional studies.

However, several limitations should be considered. Using neighborhood TB incidence as a proxy for exposure does not account for population mobility, and incomplete geocoding may have underestimated risk in the most socioeconomically deprived groups [10]. Our analysis was unable to distinguish relapse from reinfection because strain typing was not available, and important individual-level covariates, such as smoking, nutrition, occupation, and alcohol use, were not captured. The reliance on routinely collected data also introduces the potential for linkage errors and selection bias toward those accessing healthcare. While mortality data were not included here, previous analyses suggest minimal impact on recurrence estimates [9].

Finally, some of the data used are more than 20 years old, which raises questions on their reflection of current conditions. The burden of TB has changed over the past 20 years, with a peak in TB notifications in 2008 [15]. However, despite reductions in the notification rates of drug-sensitive TB since the introduction of ART, the high burden of recurrent TB still persists. We do not expect the underlying mechanisms or factors driving this burden to have changed. Therefore, the data provides valuable insight into determinants that continue to influence recurrent TB in Cape Town. We expect our results to be generalizable to similar hyperendemic settings in sub-Saharan Africa. The overall recurrent TB incidence rate in our cohort was previously estimated at 16.4 per 1000 person-years at risk (pyar) (95% CI, 16.2–16.6) (or 1.64 per 100 pyar) [9], lower than a pooled estimate of 4.10 per 100 pyar (95% CI, 2.67; 6.28) in high-incidence settings reported previously [6].

These limitations highlight key directions for future research. Prospective studies incorporating molecular epidemiology would allow disentangling relapse and reinfection. More granular spatial data and longitudinal tracking of residential mobility could refine estimates of neighborhood exposure. Finally, integrating more individual covariates, such as nutrition and smoking, through primary data collection would deepen understanding of mechanisms driving recurrent TB and could inform more precisely targeted interventions.

## 5 Conclusion

Neighborhood factors linked with increased exposure to TB (household size, incidence of TB disease) were independently associated with a higher cumulative number of recurrences, in addition to HIV infection. This suggests an important role for reinfection as an underlying mechanism. For the management of recurrent TB in Cape Town, targeted screening of neighborhoods with higher risk of recurrent TB could be explored. Such neighborhoods could be prioritized for targeted interventions.

## CRediT authorship contribution statement

**Eli Dearden:** Writing – review & editing, Writing – original draft, Software, Methodology, Formal analysis, Conceptualization. **Frank van Leth:** . **Marjan Molemans:** Writing – review & editing, Data curation. **Shumsonesa Abrahams:** Data curation. **Natacha Berkowitz:** Data curation. **Erika Mohr-Holland:** Writing – review & editing, Data curation. **Robin Wood:** Writing – review & editing, Data curation. **Sabine Hermans:** Writing – review & editing, Validation, Supervision, Methodology, Data curation, Conceptualization.

## Declaration of competing interest

The authors declare that they have no known competing financial interests or personal relationships that could have appeared to influence the work reported in this paper.

## Figures and Tables

**Table A1:** Baseline characteristics of cohorts between Secondary analysis, and Missing HIV status.

	Secondary Analysis Cohort (n = 26,851)	Missing HIV status (N = 1,990)
Male sex	13,804 (51.41)	1,144 (57.5%)
Female Sex	13,047 (48.6%)	846 (42.5%)
HIV status		
Remained negative	14,675 (54.65%)	n/a
Remained positive	12,088 (45.0%)	n/a
Seroconverted*	88 (0.33%)	n/a
Age at first episode (Years)		
0–14	4,226 (15.7%)	874 (43.9%)
15–24	5,097 (19.0%)	283 (14.2)
25–54	17,039 (63.46%)	760 (38.2%)
≥ 55	489 (1.82%)	73 (3.7%)
Mean Annual TB Incidence with standard deviation (cases per 100,000 person-years)	137.4 (84.0)	134.4 (84.4)
Mean Household size with standard deviation (# of individuals)	3.41 (0.66)	3.46 (0.7)
Mean Socioeconomic Index with standard deviation	0.43 (0.16)	0.41 (0.15)

Abbreviations: HIV: human immunodeficiency virus; TB: tuberculosis.

**Table A2:** Treatment Outcomes of Episodes 1–5 in each of the cohorts.

**Episode**	**Outcome**	**FUT10 +**	**FUT5-10**	**Secondary Analysis**
		n (%)	n (%)	n (%)
1	Completed	25,014 (77.6)	68,397 (84.0)	20,503 (83.9)
	Defaulted	3,383 (10.5)	5,645 (6.94)	1,420 (5.85)
	Died	1,648 (5.1)	3,718 (4.57)	1,293 (5.29)
	Failed	375 (1.16)	378 (0.46)	67 (0.27)
	Moved	1,389 (4.3)	2,502 (3.07)	824 (3.37)
	Not Evaluated	441 (1.37)	752 (0.92)	328 (1.34)
2	Completed	2,724 (77.0)	5,754 (81.3)	1,598 (80.4)
	Defaulted	628 (17.8)	1,062 (15.0)	307 (15.4)
	Died	20 (0.57)	17 (0.24)	3 (0.15)
	Failed	80 (2.26)	94 (1.33)	27 (1.36)
	Moved	75 (2.12)	125 (1.77)	44 (2.21)
	Not Evaluated	9 (0.25)	24 (0.34)	9 (0.45)
3	Completed	565 (71.9)	869 (72.5)	238 (70.4)
	Defaulted	179 (22.8)	278 (23.2)	85 (25.2)
	Died	2 (0.25)	0 (0)	0 (0)
	Failed	16 (2.04)	22 (1.83)	5 (1.5)
	Moved	21 (2.67)	25 (2.09)	9 (2.7)
	Not Evaluated	0 (0)	2 (0.17)	1 (0.3)
4	Completed	118 (67.1)	171 (66.8)	31 (54.4)
	Defaulted	50 (28.4)	76 (29.7)	22 (38.6)
	Died	1 (0.57)	0 (0)	0 (0)
	Failed	2 (1.14)	2 (0.78)	1 (1.75)
	Moved	5 (2.84)	5 (1.95)	2 (3.51)
	Not Evaluated	0 (0)	1 (0.39)	1 (1.75)
5	Completed	45 (59.2)	55 (54.46)	10 (43.5)
	Defaulted	26 (34.2)	43 (42.57)	10 (43.5)
	Died	0 (0)	0 (0)	0 (0)
	Failed	4 (5.26)	2 (1.98)	2 (8.70)
	Moved	1 (1.32)	0 (0)	0 (0)
	Not Evaluated	0 (0)	1 (0.99)	1 (4.35)

**Table A3:** Treatment episode intervals and mean person-years by cohort.

Treatment Episode	FUT10 + Median years (IQR)	FUT5–10 Median years (IQR)	Secondary Analysis Median years (IQR)
2nd treatment episode	1.5 (0.5–3.4)	1.1 (0.4–2.4)	0.8 (0.3–1.9)
3rd treatment episode	1.2 (0.4–2.7)	0.8 (0.3–1.7)	0.6 (0.3–1.1)
4th treatment episode	0.7 (0.3–1.8)	0.5 (0.2–1.2)	0.4 (0.2–1.0)
5th treatment episode	0.4 (0.3–0.9)	0.7 (0.2–1.2)	0.2 (0.1–1.2)
**Mean person-years (SD)**	11.4 (0.9)	7.4 (1.4)	6.3 (0.9)

**Table A4:** Reported odds ratios (95% CI) for an increasing cumulative number of TB episodes by age at first episode, across cohorts.

**Analysis**	**Age at first episode (years)**	**Episode 1 vs 2–5**	**Episode 1,2 vs 3–5**	**Episode 1–3 vs 4–5**	**Episode 1–4 vs 5**
FUT10+	0–14	0.39 (0.34–0.44)	0.20 (0.14–0.28)	0.23 (0.13–0.42)	0.12 (0.03–0.40)
	15–24	Reference	Reference	Reference	Reference
	25–54	1.02 (0.94–1.10)	0.75 (0.66–0.86)	0.62 (0.48–0.81)	0.53 (0.34–0.82)
	≥55	0.20 (0.13–0.31)	0.03 (0.005–0.21)	Unavailable*	Unavailable*
FUT5–10	0–14	0.38 (0.35–0.42)	0.16 (0.12–0.21)	0.15 (0.09–0.26)	0.03 (0.004–0.21)
	15–24	Reference	Reference	Reference	Reference
	25–54	0.98 (0.93–1.03)	0.83 (0.74–0.93)	0.72 (0.56–0.91)	0.61 (0.41–0.91)
	≥55	0.24 (0.17–0.32)	0.10 (0.04–0.26)	Unavailable*	Unavailable*
Secondary analysis	0–14	0.44 (0.38–0.53)	0.19 (0.12–0.31)	0.26 (0.11–0.64)	Unavailable
	15–24	Reference	Reference	Reference	Reference
	25–54	0.97 (0.88–1.07)	0.88 (0.71–1.10)	0.69 (0.48–0.99)	0.30 (0.14–0.68)
	≥55	0.30 (0.19–0.47)	0.16 (0.042–0.64)	Unavailable	Unavailable

**Table A5:** Comparison between the regression results of the secondary analysis cohort with and without HIV status included.

	Secondary analysis with HIV (n = 26,851)	Secondary Analysis without HIV (n = 26,851)
Variable	Odds Ratios(95% CI)	Odds Ratios(95% CI)
Neighborhood Variables		
Neighborhood socioeconomic Index*	0.93 (0.88–0.98)	0.92 (0.88–0.96)
Mean neighborhood TB annual incidence (cases per 100,000 person-years)*	1.03 (0.92–1.15)	1.08 (0.97–1.29)
Mean neighborhood Household Size*	1.50 (1.32–1.71)	1.32 (1.18–1.49)
Individual Variables		
HIV Status		
Negative	Reference	n/a
Positive	1.83 (1.59–2.10)	n/a
Seroconverted	43.7 (35.1–54.3)	n/a
Sex		
Female Sex	Reference	
Male sex	1.35 (1.24–1.47)	1.24 (1.14–1.34)
Age at First Episode (Years)		
0–14	0.47 (0.39–0.56)	0.43 (0.36–0.51)
15–24	Reference	
25–54	0.79 (0.70–0.90)	0.93 (0.83–1.04)
55≤	0.18 (0.09–0.35)	0.16 (0.08–0.32)
